# Meeting the Vitamin A Requirement: The Efficacy and Importance of *β*-Carotene in Animal Species

**DOI:** 10.1155/2016/7393620

**Published:** 2016-10-19

**Authors:** Alice S. Green, Andrea J. Fascetti

**Affiliations:** Department of Molecular Biosciences, School of Veterinary Medicine, University of California, Davis, CA 95616, USA

## Abstract

Vitamin A is essential for life in all vertebrate animals. Vitamin A requirement can be met from dietary preformed vitamin A or provitamin A carotenoids, the most important of which is *β*-carotene. The metabolism of *β*-carotene, including its intestinal absorption, accumulation in tissues, and conversion to vitamin A, varies widely across animal species and determines the role that *β*-carotene plays in meeting vitamin A requirement. This review begins with a brief discussion of vitamin A, with an emphasis on species differences in metabolism. A more detailed discussion of *β*-carotene follows, with a focus on factors impacting bioavailability and its conversion to vitamin A. Finally, the literature on how animals utilize *β*-carotene is reviewed individually for several species and classes of animals. We conclude that *β*-carotene conversion to vitamin A is variable and dependent on a number of factors, which are important to consider in the formulation and assessment of diets. Omnivores and herbivores are more efficient at converting *β*-carotene to vitamin A than carnivores. Absorption and accumulation of *β*-carotene in tissues vary with species and are poorly understood. More comparative and mechanistic studies are required in this area to improve the understanding of *β*-carotene metabolism.

## 1. Introduction

Vitamin A (VA) is an essential nutrient for all vertebrate animal species. There are two dietary sources of VA: preformed retinoids and provitamin A (pro-VA) carotenoids. The proportion of an animal's total VA supply coming from these two sources is dependent upon several major factors: (1) dietary supply; (2) intestinal absorption; and (3) metabolic ability to convert pro-VA carotenoids to retinoids. Although all carotenoids with one or more unsubstituted *β*-ionone rings can theoretically be VA precursors, *β*-carotene (BC) appears to be the most important of these. It is certainly the most studied carotenoid, at least in the context of its role as a pro-VA compound. Thus, this review will focus on the metabolism of BC across animal species. However, many of the concepts discussed here likely apply to other pro-VA carotenoids, though there is no doubt of much complexity and variation in the metabolism of those compounds as well.

A comparative approach to understanding the metabolism of BC is appropriate, as the handling of this nutrient varies impressively across animal species. Understanding how different species meet their VA requirement can inform how we feed animals that come into our care, be they production livestock, our companion pets, or exotic species kept in zoos and aquaria or managed wildlife. For this, it is important to understand the efficiency of conversion of BC to VA and how it varies in animal species. In addition, absorption into blood and accumulation in tissues of intact BC is highly variable and species-dependent. This facet of comparative BC metabolism determines to what extent a species may be impacted by the non-pro-VA functions of BC.

## 2. Vitamin A

### 2.1. Structure, Units, and Chemistry

Vitamin A is actually a family of compounds that are structurally similar to and have the essential functions and biological activity of retinol. Retinol is a long-chain, unsaturated alcohol containing five double bonds and a *β*-ionone ring. Retinol and most other naturally occurring retinoids are lipid-soluble compounds. The term “vitamin A” is often used to also include pro-VA carotenoids, but in this review, the two are distinguished and “VA” is used to exclusively mean retinoids with the biological activity of retinol. Vitamin A requirements and food values are expressed as* retinol equivalents* (RE), with 1 RE equivalent to 1 *μ*g free retinol. This conversion is important, because much of the preformed dietary VA is in the form of retinyl esters.

### 2.2. Forms and Functions of Vitamin A

**Figure 1:**
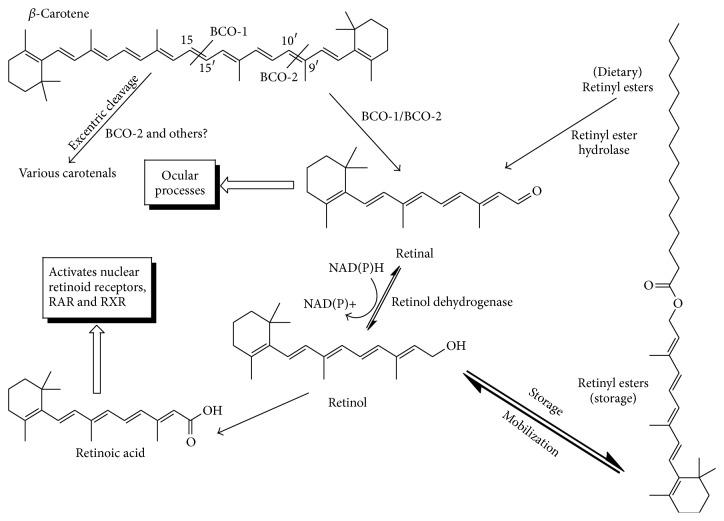
Simplified schematic of the major metabolic pathways of *β*-carotene and retinoids. BCO-1, *β*,*β*-carotene 15,15′-oxygenase; BCO-2, *β*,*β*-carotene 9,10′-oxygenase.

The most physiologically active forms of VA are retinoic acid and retinal. Retinoic acid interacts with the nearly ubiquitously expressed nuclear retinoid receptors (RAR and RXR) to regulate gene expression. Through this mechanism, VA is required for normal cellular differentiation, development, and immune function [1]. Retinal is the prosthetic group of opsins, the light absorbing units of the retina in the eye. It is thus essential for the transduction of light to the neural signals required for vision [2]. Retinol and retinal can be interconverted, and retinol can be irreversibly converted to retinoic acid (Figure 1). Retinoic acid can be utilized for most of the essential functions of VA but can not meet the requirement for vision and some reproductive functions [3].

### 2.3. Absorption and Metabolism of Vitamin A

Major sources of preformed VA include liver, fish, eggs, and, in human food systems, fortified foods such as milk, cereals, and margarine. Vitamin A is predominantly in the form of retinyl esters in foods. After release from the food matrix by digestion, retinyl esters are hydrolyzed by retinyl ester hydrolases, several of which have been described. Free retinol is taken up by enterocytes, where it is complexed with cellular retinol binding protein (CRBP-II) and then reesterified by lecithin : retinol acyltransferase (LRAT). It is then incorporated into chylomicrons and exported into the lymph along with other components of dietary fat [4]. In the circulation of humans, VA is associated almost exclusively with retinol binding protein (RBP) and is tightly regulated. However, in many other species, particularly carnivores, a significant proportion of circulating VA is in the form of retinyl esters [5]. This aspect of VA metabolism will be discussed in more depth later in this review.

If VA status is adequate, the major storage depot of VA is in the stellate cells of the liver. Hepatic storage generally accounts for 50–85% of total body VA in replete humans and is predominantly in the form of retinyl esters. Vitamin A absorption, metabolism, and storage are complex and affected by multiple proteins and control points that maintain VA homeostasis, and readers are thus referred to more complete reviews of these topics [6–8].

### 2.4. Vitamin A Deficiency and Requirements

Vitamin A deficiency results in xerophthalmia, metaplasia, epithelial differentiation, anorexia, and compromised function of both innate and adaptive immunity [4]. VA requirements have been determined using a wide range of indicators of status, including occurrence of night blindness, maintenance of normal pressure in cerebrospinal fluid, and amounts required for optimal growth and reproduction [9]. The lack of standardized indices for determination of a VA requirement makes it difficult to compare requirements across species, particularly those for which there are only one or two studies. Nevertheless, estimated VA requirements for animal species are given in Table 1.

### 2.5. Vitamin A Toxicity

Acute VA toxicity results in a range of effects within hours: general malaise, anorexia, nausea, peeling skin, hyperirritability, muscular weakness, convulsions, paralysis, and even death [10]. Chronic VA toxicity causes skeletal malformations, spontaneous fractures, and internal hemorrhage [10] and may also be linked to the development of osteoporosis in elderly humans [11]. Both VA deficiency and toxicity can cause teratogenic effects [12, 13]. Ruminants are more tolerant of high dietary VA than omnivores and nonruminant herbivores, because rumen microbes can destroy 40–70% of dietary VA [14]. Of the animal species that have been studied, adult cockatiels are among the least tolerant of high dietary VA [15]. Safe upper limits estimated for different species are summarized in Table 1.

Carnivores appear to be the class of animals most tolerant to high dietary VA. Work in the domestic cat indicates increased VA tolerance compared with other species due to the ability to increase metabolism and excretion of VA and maintain higher concentrations of circulating retinyl esters in blood [16, 17]. Cats increase urinary and fecal excretion of VA when consuming large quantities, as evidenced by the increased excretion of retinol, retinyl esters, and more polar retinol conjugates in cats dosed orally with ^3^H-retinol [16]. Retinol and retinyl esters have also been measured in the urine of the dog, silver fox, blue fox, and raccoon dog, while no VA was detected in the urine of cows, sheep, horses, rabbits, and rats [18, 19]. No more than trace amounts of VA have been observed in human urine with the exception of cases of pneumonia, kidney disease [20], or infections causing diarrhea or proteinuria [21], when retinol appears to be excreted in urine associated with RBP.

Carnivores also store tremendous amounts of VA in the liver at much higher concentrations than have been found in livers of noncarnivores. This fact is well-known among northern native people and polar explorers, who have found that consumption of polar bear and seal liver causes acute toxicity. Retinyl ester concentration in polar bear and seal livers has been found to range from 2,215 to 10,400 *μ*g/g wet weight and to vary with age and sex [22, 23]. To put this into perspective, hepatic concentrations of 20–300 *μ*g/g wet weight retinyl esters are considered to indicate “adequate” VA status in humans [4]. Similar concentrations, dependent on season, are found in herbivorous free-ranging wild species consuming natural diets [24–26]. Leighton et al. suggested that the large number of stellate cells in polar bear livers compared to other species permits the storage of high concentration of retinyl esters [23]. Species differences in hepatocyte types and proportions may help to explain differences in the ability to store large quantities of VA. Vitamin A tolerance is likely protective for wild carnivores, which may sporadically consume large amounts of VA from the liver of prey species.

### 2.6. Circulating Forms of Vitamin A in Animal Species

There are significant interspecies differences in circulating forms of retinoids. In humans, some primates, and many other omnivores and herbivores, the majority of circulating VA is in the form of retinol bound to RBP and is tightly controlled, making plasma or serum retinol nonresponsive to VA intake (unless very deficient or toxic) and not a good indicator of status [4]. In contrast, retinyl esters make up a significant proportion of circulating VA in many, but not all, carnivorous species [5]. In domestic cats, a study found 70 ± 17% of total plasma VA by weight was esterified, and of this, 61 ± 6% was retinyl stearate, 36 ± 13% was retinyl palmitate, and 5 ± 3% was retinyl oleate [18]. In a study surveying serum retinoids in 12 captive wild felid species, retinyl esters, predominantly stearate and palmitate, were detected in the serum of all species measured [27]. However, the proportion of VA present as esters ranged widely from 87% in the sand cat and 48% in the fishing cat to as low as 7% in cheetahs and cougars and 4% in servals.

In dogs, it has been shown that circulating retinyl ester concentration is proportional to dietary VA, while circulating nonesterified retinol is unaffected across a range of 210–13,200 RE/kg diet dry matter (DM) [28]. More recently, eight-week-old Labrador Retrievers and Miniature Schnauzers were fed one of four retinyl acetate concentrations to achieve intakes of 5.24, 13.10, 78.60, and 104.80 umol retinol (5000, 12 500, 75 000, and 100 000 IU VA/4184 kJ) (1000 kcal) ME for 1 yr [29]. There was no effect of VA concentration on any hematological or biochemical variables, bone-specific alkaline phosphatase, crossed-linked carboxyterminal telopeptides of type one collagen, and dual X-ray absorptiometry. Total VA concentration did have an effect on total serum retinyl esters, but there was no effect of dose on number, type, or duration of adverse events. The authors concluded that 104.80 umol retinol (100,000 IU VA/4184 KJ) (1000 kcal) ME was a suitable safe upper limit for growing dogs. Similar results were found in ferrets fed a VA supplement of 7500 RE/d as retinyl palmitate for 37 d. There was little difference in plasma retinol between the controls and the supplemented ferrets, while retinyl ester (both palmitate and stearate) concentrations increased more than 6-fold after the supplementation period [30]. Thus, it seems that dogs and ferrets are able to maintain the concentration of retinol, carried by RBP [31], within a range required for homeostasis. The retinol complexed with RBP is available for transport into cells and then interaction with nuclear receptors. The circulating retinyl esters likely represent a type of VA storage for carnivores consuming higher concentrations of VA.

## 3. ***β***-Carotene and Other Carotenoids

### 3.1. Carotenoid Structure

Beta-carotene is just one of the more than 600 naturally occurring carotenoids that have been isolated and characterized thus far [32]. All carotenoids are derived from a C_40_ polyisoprenoid skeleton, and their diversity comes from various modifications including substitution, cyclization, addition, elimination, and rearrangement [33]. They are all characterized by a long polyene chain of conjugated double bonds, called the chromophore, and this part of their structure is responsible for absorbing light in the visible region and thus producing the observed color associated with carotenoid compounds. The isomerization that can occur around each double bond contributes in part to the huge number of carotenoids, though most carotenoids in nature are predominantly present in the all-*trans* form [32]. The carotenoids are lipophilic and insoluble in water. Therefore, unless they are associated with proteins or have an additional polar functional group, carotenoids are generally located in the lipid bilayer of the cell membrane [32].

Carotenoids are divided into two groups, the carotenes and the xanthophylls. The carotenes are nonpolar hydrocarbons and include BC, *α*-carotene, and lycopene. The xanthophylls have hydroxyl or keto end groups and are thus more polar compounds, including lutein, zeaxanthin, canthaxanthin, and *β*-cryptoxanthin [34]. Because of their strongly lipophilic nature, carotenes are found in the lipid core of the cell membrane. The carotenes are also rigid molecules, so they are generally aligned parallel to the membrane surfaces. The more polar xanthophylls are more likely to expose their hydroxyl groups to the outer membrane layer, often orienting perpendicular to the membrane and at times being transmembrane in orientation [35]. These properties impact the transport, metabolism, and functions of the different classes of carotenoids. In animals, polar carotenoids seem to be more readily absorbed compared to nonpolar carotenoids [36, 37]. In humans, carotenes are more likely to be transported after absorption with LDL, while xanthophylls are evenly distributed between LDL and HDL [38, 39]. However, important species differences in lipoprotein transport may exist. For example, it has been shown in ferrets supplemented with BC that the majority of circulating BC is associated with HDL [40].

The presence of at least one unsubstituted *β*-ionine ring confers pro-VA capacity to carotenoids (Figure 1). Beta-carotene has two *β*-ionine rings, so one molecule of BC can theoretically form two molecules of retinol via retinal. In comparison, *β*-cryptoxanthin contains only one unsubstituted *β*-ionine ring (the other contains a hydroxyl group) so it can only yield one molecule of retinol. The structure of lycopene has no *β*-ionine rings so it cannot be a precursor to VA. Based on structure alone, it is thought that about 50–60 of the known carotenoids have some pro-VA activity [41].

### 3.2. History of *β*-Carotene Research

Beta-carotene was first isolated in 1831 by Wachenroder [42], who crystallized the pigment from carrot (*Daucus carota*) root and gave the compound its name. Steenbock [43] provided the first suggestion of an association between yellow carotene pigments and “fat-soluble vitamin” activity in 1919, when he made the observation that rats thrived on a diet based on yellow corn but died within 3 months when fed a diet of white corn, after developing a severe inflammation of the eyes (now known as xerophthalmia). Steenbock's work also fit with the observations of Osborne and Mendel [44], who noted that rats grew normally when the dietary fat source was butterfat but they faced “nutritive disaster” (again characterized by xerophthalmia) and ultimately death if the sole fat source was lard or almond oil. The link between BC and VA was definitively established in 1929 by von Euler et al., who demonstrated that crystallized carotene had VA activity [45]. Moore demonstrated* in vivo* conversion of BC to VA in rats in 1930 [46], and a wealth of new questions regarding the pro-VA function of carotenoids was born.

During the course of the history of the study of BC, the metabolic differences between animal species were recognized early as investigators found disparate results depending on the animal model used. For example, in his studies of BC metabolism in the early 1930s, Ahmad switched from rats to cats in search of a larger animal model, and he found that his results completely changed [47]. Amazingly, the first suggestion that the conversion of BC to VA might occur in the intestine was made during a study of baleen whales published in 1939 [48], though this speculation was not confirmed until 1947 [49]. In the 1960s, radioisotope studies measuring the absorption of BC in the lymph of rats and humans quickly determined that humans are able to absorb intact BC, while rats cannot [50, 51]. Since this time, investigators have been cautious to extrapolate observations from one species to another. In addition, the search for a better animal model for human carotenoid metabolism has led to many detailed studies in animal species, contributing to our comparative understanding of BC metabolism.

### 3.3. Food Sources of Carotenoids

Fruits such as apricots, peaches, persimmons, citrus, tomatoes, and melon are rich sources of carotenoids [52]. High concentrations are also found in green vegetables like spinach, broccoli, and parsley as well as orange tuber vegetables like carrots and sweet potatoes [52]. In addition, carotenoids accumulate in the tissues and products of animals; butterfat, egg yolk, and salmon (concentrated in the skin, muscle, and ovaries) are excellent sources [52].

### 3.4. Functions of Carotenoids

Carotenoids are not considered to be essential nutrients for animals. As long as their diets provide adequate VA, animals can live and reproduce while consuming a carotenoid-free diet without exhibiting any specific signs of deficiency. However, some free-ranging birds and fish would likely not be successful breeders without the appropriate dietary carotenoids used as pigments to communicate their viability as mates. In addition to their role as potential precursors to VA, carotenoids also have a variety of other functions that may improve animal health. Carotenoids can serve as antioxidants [53] and improve gap junction communication [54] and immune function [55]. Carotenoid intake has also been associated with reduced risk of heart disease [56], macular degeneration [57], cataracts [58], and some cancers [59, 60] in humans. In addition, BC has been shown to increase the reproductive success of cows and pigs with important implications for animal production systems [61, 62].

Unlike VA, which can be toxic at only 3-4 times the recommended daily allowance in humans, BC does not appear to have any toxicity effects [63]. As will be described in more detail later, the conversion of BC to VA is tightly regulated and dependent upon VA status. Excessive intakes of BC result in decreased efficiency of conversion to VA, thus preventing VA toxicity. This makes dietary supplementation with BC and other pro-VA carotenoids a safe way to improve VA status without risking toxicity as with retinoid supplementation, at least provided the species can utilize pro-VA carotenoids for VA. Very high intakes of BC have been known to result in harmless and transient hypercarotenemia and orange pigmentation of the skin in humans [64].

### 3.5. Digestion and Absorption of Carotenoids

Carotenoids are lipid-soluble compounds and are absorbed in the digestive tract with dietary fats. However, they must first be released from the food matrix by mastication, gastric actions, and digestive enzymes. Carotenoids in leaves and stems are usually present in the free form, while those in fruits and seeds are often esterified with fatty acids and must be hydrolyzed prior to absorption [65]. Esterified carotenoids may be less digestible than those in the free form [66], though this does not seem to be the case with lutein esters [67]. Free carotenoids in the intestinal lumen are incorporated into mixed micelles and absorbed by the mucosa of the small intestine, apparently by a passive, nonsaturable mechanism. Once in the enterocyte, carotenoids are packaged in chylomicrons and travel via the lymph to the periphery and eventually the liver. Oxidative cleavage of BC to VA occurs mainly at the brush border membrane of the intestine, with some activity in other organs such as the liver, kidney, and lungs [68].

## 4. Bioavailability of Carotenoids

Bioavailability is defined as the proportion of a consumed nutrient that is available for normal physiological functions and storage. Measuring the bioavailability of pro-VA carotenoids is made more complex by the fact that a portion of the ingested carotenoids is usually converted to VA in the intestine, before it reaches circulation. Thus, to measure true bioavailability of pro-VA carotenoids, it is also necessary to measure bioconversion to VA, which will be discussed later in this review. However, measures of apparent digestibility still provide useful information, particularly when dietary treatments are compared in the same study. In this section, the methods used to measure bioavailability and factors that have been shown to impact carotenoid absorption within a species are discussed. Discussion of interspecies differences in carotenoid absorption will be combined with a later section on interspecies differences in pro-VA activity, as these two processes interact and are difficult to separate experimentally.

### 4.1. Methods Used to Measure Bioavailability

Several methods have been used to measure carotenoid bioavailability with varying degrees of success. The simplest method involves measuring the serum or plasma response after carotenoid ingestion. This method is useful when comparing bioavailability between doses or dietary treatments; however, it can not quantify true bioavailability because it does not distinguish the dosed carotenoids from endogenous compounds or account for conversion to VA or other metabolic pools of carotenoids, such as those stored in tissues. In order to be distinguished from endogenous carotenoids, large nonphysiological doses must be used, which are likely to be less bioavailable compared with more physiological doses.

A more specific method is to measure the chylomicron response to a carotenoid dose [69, 70]. This method generates an improved estimate of absorption and distinguishes the dose from endogenous carotenoids. Because chylomicron carotenoid concentration is presumably specific to the dosed compounds and separate from endogenous pools, smaller doses can be used than with the serum/plasma response method. However, it is difficult to separate intestinal chylomicrons from liver-derived VLDL. Furthermore, carotenoids can transfer from chylomicrons to LDL or HDL and thus not be accounted for in the chylomicron response method [68].

The oral-fecal balance method can also be used to estimate bioavailability. However, this method gives highly variable results and can not account for carotenoid degradation in the upper (from chemical oxidation) or lower (from microbial degradation) gastrointestinal tract. For example, cattle given a BC preparation with rumen-protected fats had an increased plasma BC response compared with BC given with nonprotected fats [71]. It has also been shown that plant lipoxygenases present in feed plants could quickly destroy BC and lutein* in vitro* in bovine rumen fluid [72]. In addition, bioavailability of BC was higher in germ-free rats compared to those with normal gut microflora [73].

Dosing with isotope-labeled carotenoids appears to be the most promising method for determining carotenoid bioavailability [74]. An isotope-labeled dose allows absolute distinction between endogenous and dosed compounds and thus allows for the use of smaller, more physiological, doses. Some of the most informative early work on carotenoid bioavailability and conversion was conducted using ^3^H-BC. Concerns about the effects of radiation caused many investigators to turn to stable isotope applications, especially for work with human subjects. However, the impressive detection limits of accelerator mass spectrometry technology have led to a resurgence of the use of ^14^C-BC with the smallest doses yet [75, 76]. Subjects may be dosed with synthetic preparations or endogenously labeled foods grown in isotope-enriched substrates [77]. The use of isotope-labeled carotenoids determines not only absolute absorption but also postabsorptive metabolism. Thus, the conversion of BC to VA can be qualitatively measured. If a concurrent reference dose of labeled VA is given, BC conversion can also be quantified [74, 77–79].

### 4.2. Factors Impacting Bioavailability

As discussed earlier, the chemical structure of the carotenoid in question confers some inherent differences in absorption, based on chemical polarity, that impact bioavailability. Other factors affecting bioavailability include food matrix, VA status, interaction with other carotenoids, dietary fat, dietary fiber, and parasitic infection. Most of the work on bioavailability of carotenoids has been conducted in humans or rodents with several studies in ferrets. Little is known about how these factors may be similar or different in other animal species.

#### 4.2.1. Food Matrix

Bioavailability of carotenoids in raw vegetables is estimated to be 5–10% for humans and rats, while it may be as high as 50% when dissolved in oils. Thus, the food matrix has an important impact on bioavailability, and processing such as mincing, liquefying, and mild heating can improve the bioavailability of carotenoids [34, 80]. One study showed that the bioavailability of BC in spinach was improved by liquefying (9.5%) compared with whole leaf spinach (5.1%) [81]. Lutein bioavailability was measured in the same study and found to be much higher (45–55%) than that of BC and unaffected by processing. Other investigators have also observed that bioavailability of lutein in vegetables is much higher than the bioavailability of BC [37], a finding consistent with the more polar nature of lutein.

Commercially prepared water-soluble BC beadlets are perhaps the most bioavailable form of BC. In ferrets fed naturally occurring BC in carrot juice (in crystalline form in the chloroplasts), bioavailability was only about 30% of that from a water-soluble beadlet form [82]. In preruminant calves, the plasma BC response after supplementation with crystalline BC in oil was only 4% of that measured after supplementation with water-soluble BC beadlets [83].

Cooking vegetables with excessive heat (e.g., boiling) can result in degradation of carotenoids via isomerization and oxidation. While the isomers of BC may have some pro-VA activity, they appear to be less readily absorbed and/or transported compared with all-*transβ*-carotene, the predominant form in raw fruits and vegetables. It has been shown in ferrets, gerbils, and humans that the 9-*cis* and 13-*cis* BC isomers are less bioavailable than the all-*trans* isomer [84, 85].

#### 4.2.2. Vitamin A Status

Current VA status can impact BC absorption, though the data is somewhat mixed. In chicks, an inverse relationship was found between dietary VA concentration and BC absorption from yellow corn [86]. This effect was observed on apparent BC absorption determined with both the oral-fecal balance and chromic oxide methods and also in the BC content of the chicks' serum, liver, and skin. Similar results were found in a study of rats; VA-deficient rats absorbed BC at twice the rate of rats fed an adequate diet [87]. In contrast, Boileau et al. [88] observed decreased* in vitro* BC uptake by isolated brush border membrane vesicles of VA-deficient rats compared with VA-replete rats, leading these authors to postulate that VA deficiency impairs enterocyte function. Lemke et al. [76] demonstrated improved BC absorption (75% versus 55% apparent absorption) in 2 VA-replete women consuming a VA supplement for 3 weeks compared with baseline measurements. As will be discussed later, VA status also influences the efficiency of conversion of BC to VA so it may compensate for changes in bioavailability.

#### 4.2.3. Carotenoid Interactions

In humans, dietary BC appears to decrease the absorption of several other carotenoids when consumed together, including lutein [36, 89], lycopene [89], and canthaxanthin [90]. In these studies, BC absorption was not affected by the other carotenoids. On the other hand, another study [91] found that a concurrent dose of lutein (15 mg) in men decreased the absorption of a BC dose of equal mass, whereas concurrent lycopene did not impact BC absorption. In ferrets, a marked decrease in serum BC was observed following a 10 mg/kg BW dose of both BC and canthaxanthin compared with BC administered alone [92]. Interestingly, the serum canthaxanthin response was 10x lower than the serum BC response, so though canthaxanthin appeared to interfere with the absorption of BC, very little canthaxanthin itself was absorbed. These studies demonstrate that intake of other carotenoids obviously influences the absorption of BC and vice versa, but the effects may vary depending on animal species and interactions with a multitude of other factors, some described here, that impact carotenoid absorption.

#### 4.2.4. Dietary Fat

Dietary fat improves the absorption of BC, because it is required for the formation of the mixed micelles in which BC travels from the intestinal lumen into the enterocyte. In addition, fat aids in the transition of carotenoids from what is often an aqueous substrate (i.e., fruits and vegetables) to solubilization into mixed micelles. Ahmad [47] demonstrated the importance of dietary fat for absorption of BC as early as 1931 in his studies on rats. Human studies have shown that dietary fat is required for optimal absorption of BC from both supplements [64, 93] and vegetable sources [94]. However, only about 3–5 g fat may be required; increasing dietary fat above this level does not appear to improve absorption of BC in humans [95, 96].

However, there appear to be species differences in the amount of fat required for optimal BC absorption. For example, in ferrets supplemented with BC, Lakshman et al. [97] found that dietary fat concentrations of 13% or 23% improved hepatic BC storage compared with 6% fat diet. Hepatic retinol and retinyl ester concentrations were significantly improved in a dose-response manner up to the 23% fat diet, indicating that dietary fat may impact both absorption of BC and its metabolism to VA. In contrast, in horses, Kienzle et al. [98] found no difference in serum BC response to supplementation with 2.5 or 6.6% fat diets. It is possible that some species require more fat than others for optimal carotenoid absorption. From the example of ferrets and horses, one might speculate that fat is more important for optimal BC absorption in carnivores than in herbivores, which evolved consuming very low fat diets and depending on BC as an important source of VA. Regardless, most studies administer some fat with carotenoid supplements to ensure that dietary fat does not limit absorption.

Type of dietary fat may also influence BC absorption. This has been shown both* in vitro* [35, 99] and* in vivo* in rats [100, 101]. Schweigert et al. [100] found that BC accumulation in the livers of rats was higher when administered with olive or arachidonic oil compared with butter fat, lard, tallow, sunflower, soya, or linseed oils. However, sunflower oil resulted in the highest accumulation of BC in the lungs. Type of fat had little impact on plasma or liver VA. However, the animal fat sources resulted in a remarkable increase in spleen VA, up to a 10-fold increase over that observed with the vegetable fats. Thus, type of fat may influence not only BC absorption but also conversion to VA and selective tissue uptake of both BC and VA.

#### 4.2.5. Dietary Fiber

Several studies have shown that pectin, guar, and alginate reduce the absorption of BC supplements [102, 103]. Dietary fiber presumably interacts with bile acids to increase the fecal excretion of fats and other fat-soluble compounds such as carotenoids. In addition, fiber may entrap carotenoids in the intestinal lumen.

#### 4.2.6. Parasitic Infection

Parasitic infection may reduce the bioavailability of BC. A study by Jalal et al. [94] in children demonstrated increased absorption of BC from foods (mostly red sweet potatoes) when the subjects were also given anthelmintic treatment for the intestinal parasite* Ascaris lumbricoides*. Allen [104, 105] showed that chicks inoculated with coccidial infections had decreased plasma, intestinal, and liver carotenoids.

In some birds and fish, carotenoid pigmentation is thought to be an excellent indicator of fitness and is used as such by potential mates. Hamilton and Zuk [106] suggested that coloration might specifically indicate resistance to blood parasites in North American passerines. The mechanism for this could be via parasite effects on carotenoid absorption but is also likely to involve other metabolic processes required to utilize ingested carotenoids as skin and feather pigments. Negative effects of infection on pigment intensity have been reported in yellowhammers infected with* Haemoproteus coatneyi* [107], goldfinches infected with coccidiosis [108], and house finches infected with mycoplasma [109]. Milinski and Bakker [110] demonstrated that three-spined stickleback male fish have decreased intensity of red color after infection with the ciliate* Ichthyophthirius multifiliis* and are thus less likely to be selected as mates by gravid females. Similarly, Houde and Torio [111] showed that when male guppies were infected with the parasite* Gyrodactylus turnbulli*, the intensity of their yellow spots decreased, and they were avoided by females compared with their uninfected brothers.

## 5. Conversion of ***β***-Carotene to Vitamin A

### 5.1. Mechanism

The mechanism of cleavage of BC to VA has been the subject of debate since the link between the two compounds was confirmed. Early investigators recognized that there are two chemically feasible routes from BC to retinol: (1) Central cleavage of the BC molecule to yield two molecules of retinal or (2) excentric sequential oxidative attack of the double bonds of BC, eventually leading to the formation of one molecule of retinal. The central cleavage enzyme was characterized in 2001 by Leuenberger et al. [112]. They provided strong evidence that it works as a monooxygenase via a transient carotene epoxide, so the central cleavage enzyme is called *β*,*β*-carotene 15,15′-oxygenase (BCO-1; Figure 1). BCO-1 has since been cloned in Drosophila [113], mice [114], chickens [115], and human retinal pigment epithelium [116].

An excentric cleavage pathway was proposed in 1954 by Glover and Redfearn [117] and has been confirmed to occur, if at a lower rate than central cleavage. Early evidence included the appearance of some labeled apocarotenals in intestinal homogenates from humans, primates, rats, and ferrets [118]. The enzyme catalyzing excentric cleavage has since been shown to specifically catalyze the oxidative cleavage of BC at the C-9′,C-10′ double bond so it is called *β*,*β*-carotene-9′,10′-oxygenase (BCO-2; Figure 1) [119]. Most animal studies do not distinguish between the cleavage activities of BCO-1 and BCO-2; therefore, we use the more generic term “BCO” to describe enzyme activity measured as the formation of VA.

Conversion of BC occurs most efficiently in the intestine. In rats, Duszka et al. [120] found maximal activity of BCO in the jejunum and noted that activity was much higher in mature functional cells than in stem cells. BC cleavage activity has also been described in liver, lung, kidney, and brain tissues of the rat, though these sites are quantitatively much less important than the intestine [121]. The activity of BC metabolizing enzymes may change with development. Yamaguchi et al. [122] showed that in prehatch chicks, there was no intestinal BCO activity but significant hepatic activity. Posthatch, intestinal cleavage activity was rapidly induced within 24 hr, while activity in the liver decreased.

### 5.2. Methods Used to Measure *β*-Carotene Conversion

Several methods have been used for measuring BC conversion or biopotency relative to preformed VA. It is important to point out that each method has its pitfalls and that each gives a slightly different type of answer to the same question. This makes it difficult to compare data from studies using different methods to measure BC conversion. The chylomicron response method and isotope-labeled dosing methods were discussed in the section on methods for measuring absorption of BC. Two other methods have been used to measure conversion of BC to VA and are worth mentioning here, the functional bioefficacy method and enzyme activity assays.

The functional bioefficacy assay was used in some of the early studies in animals [123–125] and humans [125] and contributed much to the understanding of the quantitative value of BC as a source of VA. It is still used frequently with animals. With this method, study subjects first must be depleted of VA stores and verified as such through measurement of liver VA concentration or clinical signs of VA deficiency (dark adaptation was used in the human studies). Groups of subjects are then repleted with different amounts of preformed VA (usually as retinyl acetate or palmitate) or BC (either as a synthetic supplement or as contained in food). VA repletion is determined by measuring liver stores or other functional indices, and regression lines for VA status and both dietary VA and BC are calculated. The ratio of the regression coefficients of the BC line to the VA line gives the potency of BC as a source of VA. The advantage of the functional bioefficacy method is that it is a direct measurement of the utilization of BC, taking into account bioavailability of the BC source. On the other hand, this method does require VA depletion, which can take several years in many animals and may be ethically objectionable. In addition, accurate assessment of depletion and repletion requires measuring hepatic VA stores, which is an invasive procedure. Another disadvantage to this method is that BC conversion to VA has been shown to be more efficient in VA-deplete subjects than in replete subjects [87, 126, 127], so BC conversion efficiency may be underestimated with this method compared to conversion efficiency in normal replete subjects.

Enzyme activity assays have been used with tissue homogenates to determine the rate of conversion of BC to VA in a variety of species [120, 128–131]. Though this method gives quantitative results, the* ex vivo* nature limits the application of the data to calculation of the contribution of dietary BC to the VA supply. Assay conditions are unlikely to exactly mimic the tissue environment, particularly given the many nutrient interactions that can impact BC metabolism. In addition, reaction conditions have been modified over the years, making it difficult to compare enzyme activity measurements from different studies. Enzyme assays are most useful for determining that an animal is capable of conversion and comparing the enzyme activity of tissues homogenates isolated from different animals species under the same assay conditions.

### 5.3. Effect of Vitamin A Status on *β*-Carotene Metabolism

One characteristic of BC metabolism to VA is that its conversion efficiency is affected by VA status. VA-deficient animals are more efficient at converting BC to VA than replete counterparts. Conversely, with increasing consumption of BC, cleavage to VA becomes less efficient, making BC a safe supplement without risking VA toxicity. VA-deficient rats have been shown to have greater intestinal BCO cleavage activity [87, 126] and increased plasma VA in response to a BC dose [126]. In a human study, Ribaya-Mercado et al. [127] demonstrated that Filipino children with poor VA status were more responsive to a dietary BC intervention than those with better baseline VA status.

Some insight into the mechanism of the homeostatic effects of VA status on BC metabolism was provided by James and El Gindi [132]. These authors showed that the activity of the intestinal BCO enzyme in rats decreased* in vivo* in a dose-dependent matter with oral doses of retinyl acetate, BC, and retinoic acid and increased with the administration of a RAR*α* receptor antagonist. They also reported that treatment of VA-deprived chickens with retinoic acid resulted in a significant decrease in intestinal BCO mRNA. Thus, these authors suggest that retinoids and carotenoids regulate intestinal BCO activity at the transcriptional level via RAR interactions.

### 5.4. Other Dietary Factors Impacting *β*-Carotene Metabolism

Both the type [132] and dietary concentration of protein [133] have been shown to influence BC metabolism in rats, with moderate to high levels of protein improving VA formation. Higher fat diets appear to promote BC conversion to VA in gerbils [134], ferrets [97], and rats [121]. Total carotene load has an inverse effect on BC utilization such that conversion is most efficient when BC is fed in small amounts, on the order of 1-2x that needed to meet the VA requirement; above this, conversion efficiency declines [135].

## 6. Species Differences in ***β***-Carotene Metabolism

Animal species handle BC differently with respect to absorption, accumulation in blood and tissues, and metabolism to VA. Although BC metabolism has been studied in many species, the variability in methods used makes it difficult to make quantitative interspecies comparisons. However, some patterns emerge. In the following section, mammals are classified as herbivores, omnivores, and carnivores, and the research on members of each group is reviewed. Birds and fish are also discussed separately, as their metabolism of carotenoids and retinoids is distinctive from land mammals in several respects. Reviewed data are also summarized in Table 2 of conversion efficiency ratios and Table 3, a summary of absorption, tissue accumulation, and conversion to VA in animal species.

### 6.1. Herbivorous Mammals

#### 6.1.1. Cows

Cows absorb intact BC into circulation, accumulate it in tissues, and utilize BC as an important source of VA. In one study evaluating VA (retinol and retinyl esters) and BC in 59 different mammalian and bird species, animals in the order Artiodactyla (cows and gaurs) were one of the only orders to have BC detectable in plasma [136]. Mora et al. [137] compared plasma and tissue concentrations and intestinal BC cleavage activity in cattle and goats. In cattle, plasma BC concentrations reflected dietary intake. Tissue concentrations of BC in cattle are highest in the corpus luteum [138]. There was significant BC cleavage activity measured in both cattle and goats, though goats had higher activity of the two species [137]. BC conversion to VA in bovine intestinal mucosa isolates has been confirmed in several other studies [128, 137].

Absorption of BC and conversion to VA may vary with cattle breed; it has been noted that Holsteins have less pigmented adipose and milkfat, while Jerseys and Guernseys have yellow adipose and milkfat [9]. Morales et al. [139] found no difference in intestinal and hepatic BCO-1 mRNA expression between cattle with pigmented or less pigmented adipose tissue. There was also no difference in the intestinal BCO enzyme activity between pigmented and less pigmented cattle, though hepatic BCO activity was higher in pigmented cattle. This study did not report the breed or the diets of the cattle; however, it does indicate that adipose pigmentation occurs independently of intestinal BCO activity and is not prevented by increased BCO activity in the liver.

Supplementation with BC has been shown to improve reproductive performance in cattle [61], and BC cleavage to VA in reproductive tissues may play a role in the mechanism of this effect. BCO activity has been measured in bovine ovarian follicles, and a significant positive correlation between follicle maturation and both BC conversion rate and vitamin A concentration in follicular fluid was found [129]. BC cleavage activity has also been measured in the corpus luteum of cattle; activity at mid-ovulation was 2x that measured in intestinal homogenates [140]. Oral supplementation with 2000 mg of BC daily from d 21 before the expected calving date until parturition supported the onset of luteal activity during early lactation in dairy cattle [141]. The dose of BC in this study approximated the amount of intake at grazing during the close-up dry period.

In preruminant calves, oral supplementation with BC results in a plasma BC response [80, 83, 142, 143] as well as increases in tissue BC concentration [80, 143]. Hoppe et al. [144] found a dose-response relationship between dietary BC and plasma, liver, and perirenal BC and hepatic VA concentrations, providing evidence that calves both absorb BC intact and convert it to VA. Kon et al. [145] found that calves converted orally administered BC to VA in blood and liver, while an intravenous injection of BC did not result in increased VA. The same study found that rats and rabbits could utilize injected BC for VA, so these authors speculated that the lack of extraintestinal metabolism in calves may be at least partly responsible for the accumulation of BC in adipose of cows. However, this is not supported by the previously mentioned work of Morales et al. [139].

Slifka et al. [146] measured serum carotenoids of several more exotic members of subfamily Bovidae Bovinae from zoo collections, including a wisent and banteng, and found relatively high circulating concentrations of BC. However, cattle and their close relatives appear to be unique among the species of the family Bovidae, many of whom have been designated nonaccumulators of carotenoids despite consuming moderate to high dietary levels (see “other Artiodactyla”). BC can be destroyed by rumen bacteria [72] and lipoxygenases present in some forage plants [73], and these factors may contribute to variability of responses to BC consumed by cattle and other herbivores.

#### 6.1.2. Sheep and Goats

Sheep and goats do not appear to readily absorb intact BC but are efficient convertors of BC to VA. Sheep and goats have colorless fat, indicating that they do not accumulate BC in their adipose tissue [147]. Apparent absorption of BC was measured in sheep using the oral-fecal balance method [148]. Sheep were dosed daily with 2.9–8.5 mg BC in alfalfa pellets or a water-soluble synthetic solution, and 90–98% of the dose was recovered in the feces. When the dose was dissolved in fat, fecal recovery was reduced to 50%, but the authors found that this was due to BC destruction in the lower intestinal tract, not increased absorption. In goats supplemented with very high levels of BC, BC did not appear in serum until after 10 days of supplementation [137].

Martin et al. [123] conducted a functional bioassay experiment measuring the VA activity of pro-VA carotenoids (predominantly BC) from corn silage in lambs. By measuring serum and hepatic VA, they determined that, in VA-depleted lambs, 7.4 *μ*g BC = 1 *μ*g retinol (RE). Yang and Tume [128] measured BCO activity in isolated intestinal mucosa of sheep and found a specific activity of 13 pmol BC cleaved/mg protein/hr. Enzyme activity of goats and cattle was measured under the same conditions and found to be 5.5 and 4, respectively. Mora et al. [137] also measured BC cleavage activity in both goats and cattle and found goats to have higher enzyme activity than cattle. BC is no doubt a critical source of VA in all three herbivorous species, but sheep and goats may convert BC to VA at a higher rate than cattle.

#### 6.1.3. Other Artiodactyla

Artiodactyla comprise a large group of animals, all even-toed ungulates. Artiodactyla include cows, sheep, goats, and pigs, which are all discussed in separate sections. Serum carotenoids have been measured in several other Artiodactyla species, and they bear mentioning because carotenoids are remarkably absent from their serum, despite their herbivorous dietary habits (i.e., high carotenoid, devoid of preformed VA). Slifka et al. [146] found no detectable BC in the serum of pygmy hippos, Bactrian camels, Pere David's deer, okapi, giraffe, sitatunga, klipspringer, eland, kudu, Congo buffalo, and Siberian ibex. Moore [147] also noted that white-tailed deer have colorless adipose tissue. Thus, among the Artiodactyla, cows and their close relatives seem to be distinguished by their accumulation of BC in plasma and tissues. However, the herbivorous dietary habits of most Artiodactyla must require that they successfully convert BC to VA in order to meet their VA requirements.

#### 6.1.4. Rabbits

Rabbits have colorless fat so they are considered nonaccumulators of BC [147]. Several studies have measured BC cleavage activity in the intestinal mucosa of rabbits [130, 131]. In the comparative study of enzyme activity by Lakshmanan et al. [130], the specific activity of BC cleavage in rabbits was similar to that in the chicken, a tortoise species, and a monkey species, but less than that measured in the guinea pig or a freshwater fish. Consistent with their herbivorous diet, BC is likely the sole source of VA for rabbits.

#### 6.1.5. Horses

Horses have yellow fat so they seem to accumulate BC in adipose [147]. Kienzle et al. showed a significant increase in serum BC when horses were supplemented with either a water-soluble beadlet preparation or grass meal containing BC, with no observed difference in response between the two BC preparations [98]. These authors also observed that there was no difference in serum response when supplements were given with diets with 2.5 or 6.6% fat, contrary to results in rats [47], ferrets [97], and humans [64, 94, 95]. Fonnesbeck and Symons [149] showed that plasma BC was correlated to dietary BC intake from several forages with varying concentrations of BC. In addition, horses fed alfalfa, which had the highest BC concentration of the forages tested, had higher plasma retinol compared to those consuming the other diets. Relatively high concentrations of plasma BC have also been measured in free-ranging Przewalski's horses [150].

Greiwe-Crandell et al. [151] supplemented VA-depleted horses with 215 mg of BC in synthetic beadlet form daily for 4 weeks and found that VA status, measured by serum retinol and a relative dose-response test, was not improved as compared to controls and horses supplemented with 22 mg retinyl palmitate. The retinyl palmitate dose was twice that recommended by the NRC. The inefficacy of the BC supplement in this study may indicate that horses require more than 10 *μ*g of BC to form 1 RE, the current NRC estimate for horses. Regardless of their conversion efficiency, the herbivorous dietary pattern of horses indicates that they must utilize carotenoids to meet their VA requirement, as their natural diet contains no preformed VA.

### 6.2. Omnivorous Mammals

#### 6.2.1. Rodents

Some the first work on BC metabolism was done in rats. Studies dosing rats with ^14^C-BC demonstrated efficient conversion of BC to retinol in rats but showed that rats do not absorb intact BC into blood [50, 152, 153]. On the other hand, Kon et al. [145] injected rats intravenously with BC preparations and were able to measure BC in both plasma and liver. There was also evidence that the rats converted injected BC to VA, presumably extraintestinally. Thus, though rats do not normally absorb intact BC, they apparently have the ability to accumulate and utilize it for VA should it enter the bloodstream. Rats dosed with very large quantities of BC (175 mg 2x/week for 4 weeks) did accumulate it in liver and lung tissues, but BC was not detectable in plasma [100]. BC cleavage activity has also been demonstrated in intestinal homogenates from rats [120, 154] and guinea pigs [131, 155].

The conversion ratio in rats has been estimated to be 2 *μ*g BC = 1 RE [135, 156], though Marusich and Bauernfeind measured a conversion ratio of 6–10 *μ*g BC (water-soluble beadlet form) = 1 RE based on hepatic VA stores using a functional bioassay model; the higher end of the conversion ratio was for higher levels of BC supplementation [157]. Brubacher and Weiser noted that the conversion ratio may only be as low as 2 *μ*g BC = 1 RE when rats are fed just enough BC to meet their requirement (<0.3 *μ*g/kg BW) [135].

Unlike rats, Mongolian gerbils absorb dietary BC and accumulate it in tissues [134, 158, 159]. Lee et al. [158] estimated the VA equivalency of BC to maintain tissue VA in gerbils at 6–13 *μ*g BC = 1 RE.

#### 6.2.2. Humans

Humans are among the most studied animals with regard to BC metabolism, likely because rats were quickly proven to be unsuitable models because of their inability to absorb BC. Other animal models have been proposed [159], but the use of isotope-labeled BC has enabled detailed noninvasive metabolic studies in humans. These have recently been reviewed [74, 160, 161], so this review will only briefly summarize the data on BC metabolism in humans. BC is readily absorbed in the blood of humans [51, 162] and is found in circulation of humans consuming a wide range of omnivorous diets [163] with correlations between intake and circulating concentrations [164]. Humans also have yellow fat [147], so it is assumed that they accumulate BC in adipose. BC can fulfill the VA requirement for humans, though early estimates of conversion efficiency were based on a depletion-repletion model so it may have overestimated conversion efficiency [165]. According to the Institute of Medicine's current recommendations, the conversion ratio is estimated at 2 *μ*g pure BC in oil = 1 RE and 12 *μ*g BC in food matrix = 1 RE [166]. The study of van Lieshout et al. [79] suggests that estimated conversion efficiency of BC in oil is somewhat inadequate; these authors found 2.4 *μ*g BC in oil = 1 RE in Indonesian children. Some studies have found even higher conversion ratios; for example, ratios of 9.1 *μ*g BC in oil = 1 RE in adults [78] and 15 and 21 *μ*g BC in carrots and spinach = 1 RE, respectively [77]. Thus, the conversion ratio for BC in humans continues to be the subject of much debate and of great interest because of its role in meeting the VA requirement of people in developing countries, where VA deficiency continues to be a problem.

#### 6.2.3. Nonhuman Primates

Similar to humans, the nonhuman primates that have been studied also seem to absorb BC intact into blood. This has been demonstrated in rhesus macaques [152] and squirrel monkeys [163, 167]. The studies on squirrel monkeys demonstrated selective absorption of carotenoids, with zeaxanthin preferentially absorbed compared with BC. García et al. [168] measured carotenoid concentrations in great ape diets and plasma and noted selective accumulation of lutein in plasma compared with higher dietary concentrations of BC. Krinsky et al. [152] dosed rhesus macaques with ^14^C-BC and noted a strong response of radioactive retinol in both serum and liver. Intact ^14^C-BC was also found in serum, liver, colon, heart, kidney, lungs, ovary, pancreas, intestine, spleen, and stomach. BC cleavage activity has also been measured in intestinal homogenates of primates [118, 130].

#### 6.2.4. Pigs

Pigs have white adipose tissue [147] and are assumed to be low absorbers and accumulators of BC. Schweigert et al. [169] was able to demonstrate low concentrations of BC in the plasma, liver, adrenals, and corpora lutea of pigs supplemented with high concentrations of BC (100 mg BC/kg diet for 14 weeks). From hepatic VA stores, the authors of this study estimated a conversion ratio of 40 *μ*g BC = 1 RE, though this low conversion ratio was likely inflated by the high dose of BC used. In pigs dosed with 30 mg ^14^C-*β*-carotene, Schweigert et al. reported that labeled BC was detected in both the colon and lung, though not in the plasma, liver, kidney, or intestine [170]. These authors also demonstrated conversion of ^14^C-BC to labeled retinol, though only 4% of the labeled BC dose was recovered as retinol. BC cleavage activity has also been detected in intestinal homogenates isolated from pigs [155, 171].

Several earlier studies examined the efficiency of utilization of BC for VA in pigs using a functional bioassay model. These found that the VA equivalency of BC from corn ranged in 13–27 *μ*g BC = 1 RE [124, 125, 172], a somewhat more efficient estimate than that from Schweigert et al. and likely more accurate since they used more physiological supplementation rates of 1–10 mg BC/kg diet. Regardless, pigs seem to be relatively inefficient at both absorbing BC and converting it to VA compared with other omnivores studied.

### 6.3. Carnivorous Mammals

#### 6.3.1. Canids

Dogs have been found to have no to moderate concentrations of BC in circulation when consuming unsupplemented omnivorous diets that likely contain some carotenoids [173–176]. Dogs have colorless fat [147], so it appears that they do not accumulate BC in their adipose. Chew et al. [175] studied the plasma response to oral BC supplementation in beagle dogs. Unsupplemented dogs consuming a commercial canine maintenance diet had no measurable BC in plasma. When dogs were given single oral doses of 50, 100, and 200 mg of BC in water-soluble beadlet preparation, plasma BC response was significant and concentration increased in a dose-dependent manner. Plasma BC also accumulated in plasma, lymphocytes, and neutrophils when dogs were dosed for up to 30 days with BC [175]. However, the plasma response to the BC dose was much lower in dogs compared to similar single dose studies in cats [177], ferrets [178], and calves [142, 143].

Crissey et al. [176] analyzed serum from 6 captive wild canid species (African wild dog, arctic fox, gray wolf, maned wolf, Mexican wolf, and red wolf) and found no detectable carotenoids in any samples. Their analysis included BC, lutein, *β*-cryptoxanthin, lycopene, and *α*-carotene. Though the diets of these animals were not analyzed for carotenoids, the dietary composition of the maned wolves in this study included 21% fruit and 21% vegetables so they would have moderate concentrations of carotenoids. Slifka et al. [146] also studied grey wolves and cape hunting dogs consuming zoo diets with moderate to high carotenoid concentrations and found no detectable carotenoids in serum.

In 1934, Turner [179] demonstrated that dogs can utilize BC to meet their VA requirement. Dogs were fed a basal diet of meat and boiled rice and were either not supplemented or supplemented with 150 g fresh carrots with and without an additional 10 mL cod liver oil. The two unsupplemented dogs died after 15 and 49 days. The supplemented dogs from both treatments apparently remained healthy throughout the experiment. After 63–224 days on the supplemented diets, there was no difference in hepatic or renal VA concentration between the dogs receiving the cod liver oil or not. Several years later, Bradfield and Smith [180] confirmed this finding, showing no difference in hepatic VA between dogs supplemented with 200 IU of cod liver oil, BC in oil, or BC from carrots, though it is unclear how the authors defined IU units of BC. This definition would have required some assumption about the conversion efficiency of BC to VA. Thus, quantitative conversion efficiency has not been estimated in dogs, though it is clear that dogs both absorb BC and can utilize it as their sole source of VA. It has also been shown that foxes can utilize BC as a source of VA [181]. Dogs seem to be more efficient at converting BC to VA than either ferrets or felids, consistent with their more omnivorous diet compared to the strict carnivores.

#### 6.3.2. Ferrets

Ferrets have been proposed as an animal model for human BC metabolism and have been studied in some detail. Ferrets absorb dietary BC into the blood and accumulate it, though at very different concentrations, in several tissues, including liver, adipose, lung, spleen, kidney, muscle, and skin [82, 92, 178, 182]. Lederman et al. [183] determined in a functional bioassay (based on hepatic VA) that ferrets do convert BC to VA, but their conversion is inefficient, estimated at 15 *μ*g BC < 1 RE. On the other hand, BCO enzyme activity has been successfully measured in several ferret tissues, including intestine, liver, lung, and adipose tissue [118, 184].

#### 6.3.3. Felids

The absolute requirement for preformed VA and inefficacy of BC was one of the first metabolic idiosyncrasies of domestic cats to be discovered. Ahmad [47] was unable to detect the conversion of BC to VA in liver perfusion or oral dosing experiments with VA-deficient cats. In the experiments of Gershoff et al. [185], most VA-deficient cats did not respond to IV or oral doses of BC, though 1 of the 10 cats tested with an oral dose did show a slight increase in serum VA and BC. Lakshmanan et al. [130] tested intestinal homogenate from cats (perhaps just one, number not stated) and found no measurable BC cleavage activity. Many of these early studies were conducted before the advent of HPLC methods for analysis of retinoids and carotenoids so they suffered from a lack of sensitivity and specificity of analytical methods. Data from our laboratory indicates that cats do have the ability to convert BC to VA, though the conversion efficiency is very low [186]. Regardless, it is clear from the earlier depletion studies that BC cannot replace VA in the diets of cats.

Some early studies were unable to detect absorption of orally administered BC into blood or the liver of domestic cats [47, 185]. However, more recent studies and our own experiments have found absorption of BC supplements into plasma to be quite substantial in cats, though these studies use relatively high doses [177, 186, 187]. Domestic cats consuming commercial diets seem to have very low to no circulating BC [18, 187]. However, Crissey et al. [27] found relatively high concentrations of serum BC in 11 captive wild felid species kept in zoos. Dietary carotenoids were not quantified in this study, but regardless, wild felids seem to readily accumulate BC in blood.

### 6.4. Birds

Xanthophylls are the most important class of carotenoids for pigmentation in birds, and these are absorbed preferentially over the carotenes and give the bright colors to beak, feathers, legs, and yolk. For example, Capper et al. [188] found that the liver oil of hens on a normal diet (with “greenstuffs”) was a deep yellow color, while the liver oil of hens fed a purified diet supplemented with BC was only pale yellow in color. Chickens are generally thought to be poor absorbers and accumulators of carotenes [189]. Chicken yolk is very low in BC, instead having lutein and zeaxanthin as the dominant carotenoids, even when hens are supplemented with dietary BC [190]. A recent study determined that yellow skin color in chickens, the most abundant phenotype, is caused by a regulatory mechanism that inhibits the expression of BCO-2 in the skin. BCO-2 asymmetrically cleaves BC and other carotenoids to colorless apocarotenals and retinal, so its inhibition leaves the colorful carotenoids as skin pigments [191].

While chickens are the most studied of the birds, there is likely great diversity in carotenoid metabolism in other birds. For example, unlike chicken egg yolks, the egg yolks from several wild birds (gulls, coots, and moorhen) were found to contain high concentrations of BC, about 25–30% of total carotenoids [192]. Gulls were also found to have high concentrations of BC in the liver [193]. On the other hand, Slifka et al. [146] measured serum carotenoids in a variety of bird species kept at a zoo and found no detectable serum BC in any of the species studied (included greater flamingo, American flamingo, Mandarin duck, grey gull, scarlet ibis, Inca tern, wood duck, hybrid teal, Hadada ibis, whistling duck, brown pelican, sacred ibis, Humboldt penguin, and Brazilian teal). Canthaxanthin was present in high concentrations in the serum of the flamingos, while lutein + zeaxanthin (coeluting on HPLC) were high in the serum of the remaining species.

Birds do utilize BC as an important source of VA. Several functional bioassays in chickens have demonstrated that BC can replace preformed VA in the diets of chickens [157, 188, 194], and the NRC settled on a conversion ratio of 2 *μ*g BC = 1 RE [195], implying that chickens, along with rats, are among the most efficient animals at converting BC to VA; BC cleavage activity has also been measured* ex vivo* in the intestinal mucosa of chickens [196, 197]. The utilization of BC to meet the VA requirement has also been shown in cockatiels [198], canaries [199], bobwhite quail, and ducks [200]. The data on cockatiels indicates that they are somewhat less efficient than chickens, while canaries appear to be equally efficient as chickens at utilizing BC for VA.

The utilization of BC has not been investigated in carnivorous birds. However one study did report retinol concentrations in birds were higher compared to mammals. Retinyl esters (retinol palmitate and oleate) represented 10–50% of VA in birds of the order Ciconiiformes and Falconiformes [136]. The occurrence of blood VA esters in these two orders of birds and carnivorous mammals may represent an adaptation to a carnivorous diet with a high supply of VA [136].

### 6.5. Fish

VA exists in two major forms in fish: as retinol, also known as A_1_, and as 3,4-didehydroretinol, also known as A_2_. A_2_ differs from retinol in that it has an additional double bond between carbons 3 and 4 in the *β*-ionone ring. A_2_ is part of the visual pigment porphyropsin and accompanies the retinal protein rhodopsin in the eyes of teleost fish as well as in reptiles, amphibians, and crustaceans [201]. In general, A_2_ is the predominant retinoid and visual pigment in freshwater fish, while retinol dominates in marine fish [202], though there appear to be some exceptions to this rule [201]. Retinol can be converted into A_2_ but not vice versa [203]. The respective roles and relative potencies of retinol and A_2_ for biological functions in fish are not well understood. However, A_2_ appears less potent than retinol when it comes to meeting the VA requirement in mammals. The biological activity of A_2_ was measured in rats with a growth assay and was found to be 40% of pure retinol [204]. Recently, Kongsbak et al. [205] reported on the effects of a dietary intervention in Bangladeshi children using a small native freshwater fish,* Amblypharyngodon mola*, found to contain predominantly A_2_, and concluded that this was a poor source of VA for children.

Fish generally absorb and accumulate dietary xanthophylls more efficiently than carotenes [201]. Several studies have demonstrated efficient conversion of BC and other carotenoids to VA. These studies are generally qualitative in nature and do not attempt to estimate conversion efficiency. Investigators reported difficulty with administering a quantitative dose of carotenoids to fish; much of the dose ended up in the water [206, 207]. Barua and Goswami administered BC and lutein to depleted* Saccobranchus fossilis*, an Indian catfish. About four hours after the dose, significant quantities of retinoic acid were recovered from the intestines of the fish in almost all cases, with retinol appearing in just a few cases [206]. On the other hand, administration of lutein resulted in almost exclusive recovery of A_2_ [206, 207]. Goswami [203] showed in several fish species that *β*-cryptoxanthin was converted to retinol in fish that naturally accumulate more retinol and to A_2_ in fish that naturally contain more A_2_. Gross and Budowski [208] reported that guppies and platies form VA (predominantly retinol with some A_2_) from BC, isozeaxanthin, canthaxanthin, and astaxanthin. They found that lutein administration resulted in only a very small increase in A_2_ in these species. Schiedt et al. [209] confirmed that astaxanthin, canthaxanthin, and zeaxanthin were all VA precursors in VA-deficient rainbow trout but that pro-VA activity was significantly decreased in VA-replete fish. The authors did not administer BC in this study, but they suggested that it must be an intermediate in the pathway from xanthophylls to VA [201, 209].

In summary, the literature demonstrates that several carotenoids are important precursors to both retinol and vitamin A_2_ in fish. Which form of VA is produced from pro-VA carotenoids seems to be species-specific. Like in mammals and birds, pro-VA conversion efficiency appears to be regulated by VA status. However, fish may be distinct in having reductive pathways from xanthophylls to BC and lutein to retinoids.

## 7. Conclusion

The metabolism of BC has proven to be an endless field of study with metabolic variations being equal to the number of animal species studied. Many factors impact the bioavailability of BC and its conversion to VA, and estimation of the value of BC in mixed diets and under various conditions is difficult. Nevertheless, some patterns emerge (Tables 2 and 3). Several omnivores (rat and chicken) appear to be the most efficient converters of BC to VA. Perhaps this is due to the diversity of potential diets in these omnivores, requiring them to be the most flexible and able to readily utilize BC or preformed VA when available. Herbivores also have high conversion efficiency, though they likely do not require maximal BC conversion efficiency since their diets generally have high concentrations of pro-VA carotenoids. It is not surprising that carnivores are less efficient at converting BC to VA, as they evolved consuming diets with abundant preformed VA. It would be useful to conduct quantitative studies of BC conversion in more animal species to better define the spectrum of conversion efficiencies and determine if this general pattern (omnivore > herbivore > carnivore) remains. In particular, only a few species of carnivores have been studied. Domestic cats are often assumed to be a good model species for all carnivores, but the example of dogs and ferrets demonstrates that there is likely a spectrum of BC conversion efficiencies even within carnivores. In addition, there is no data on BC conversion on other faunivores, such as those that eat insects or other invertebrates, foods that have very different concentrations of preformed VA and carotenoids than vertebrate tissue.

The variability between species in the ability to absorb BC intact and accumulate it in tissues is not understood. Early observations that some species had yellow adipose and others had white adipose led investigators to speculate that there was a relationship between conversion efficiency and tissue accumulation. It was thought that white adipose species are perhaps so efficient at intestinal cleavage of BC that very little escapes into circulation. However, the story seems to be more complex. Some white adipose species are relatively poor convertors but do absorb BC into circulation (e.g., cats and ferrets) and among the most efficient convertors are species with both white (e.g., sheep, goats, rats, chickens) and yellow adipose (e.g., cows and humans). Why are there species differences? Little research has been conducted in this area. It is often said that BC absorption occurs via a passive mechanism, but the selective absorption of carotenoids in many species indicates this may not be the case. Some investigators have proposed that there are specific carrier proteins in the gut and other tissues that may regulate the absorption and accumulation of carotenoids [210–222]. Accumulation of carotenoids in the tissues of some species but not others may be related the lack of extraintestinal BCO activity, as has been proposed in cows [145]. In chickens, the inhibition of the expression of BCO-2 in the skin allows for the accumulation of carotenoids in that tissue [191]. Thus, species- and tissue-specific accumulation may be related to the expression and inhibition of BCO enzymes. Perhaps examining BCO activity beyond the gut would improve our understanding of species differences with regard to BC accumulation. Absorption and accumulation of intact BC in animal species determines if BC may have important functions (i.e., antioxidant, immune response, and gap junction communication) beyond its role as a VA precursor.

## Figures and Tables

**Table 1:** Estimated vitamin A requirements and safe upper limits in RE^1^/kg dry matter in animal species. Requirements listed are the minimum requirements or adequate intake values.

Species	Physiological state	Requirement	Upper limit	References
Cat	Growth	1000	80,000	[210]
	Maintenance	1000	100,000	
	Gestation and lactation	2000	100,000	

Catfish	All	450	9999	[10, 202]

Chicken	Growth	450	4500	[10, 195]
	Laying	900	13500	

Cockatiel	Maintenance	600	<3000	[15]

Common carp	All	1200	ND	[10, 202]

Cow, beef	Feedlot	660	19800	[10, 211]
	Pregnant heifers and cows	840	19800	
	Lactating cows and bulls	1170	19800	

Cow, dairy	Growth	1014	19800	[10, 212]
	Lactating cows and bulls	840	19800	

Dog	Growth, gestation, lactation	1212	15,000	[210]
	Maintenance	1212	64,000	

Fox	Growth	732	ND	[213]

Geese	Growth	450	4500	[10, 195]
	Breeding	1200	4500	

Goat	Maintenance	1500	13500	[10, 214]
	Lactation	1750		

Hamster	All	1223	ND	[156]

Horse	Maintenance	549	4800	[215]
	Growth, working	450–690	4800	
	Pregnancy and lactation	825–1110	4800	

Human	Adult male	1200	6000	[10, 160]

Mink	Growing	1779	ND	[213]

Mouse	All	800	ND	[156]

Pig	Growing, 5–10 kg	660	6000	[10, 216]
	Growing, 20–120 kg	390	6000	
	Pregnant swine and boars	1200	12000	
	Lactating	600	12000	

Rabbit	Growing, maintenance	174	4800	[10, 217]
	Gestation	497	4800	

Rainbow trout	All	750	7500	[10, 202]

Salmon	All	750	7500	[10, 202]

Sheep	Replacement ewes, 60 kg	470	13500	[10, 218]
	Pregnancy, 70 kg	992	13500	
	Lactation, 70 kg	714	13500	
	Replacement rams, 80–100 kg	593	13500	

Turkey	Growing and breeding	1500	4500	[10, 195]

^1^1 retinol equivalent (RE) = 1 *μ*g retinol.

**Table 2:** Estimated efficiency of conversion of *β*-carotene to vitamin A in animals, defined as *μ*g of *β*-carotene required to form 1 RE (1 *μ*g retinol). The *n* value given is the total number of animals in the study.

Species	Conversion ratio	Study design	References
Herbivorous mammals			
Cow	8.33	Recommendation extrapolated from study in lambs [122].	[123, 212]
Sheep	5.56–8.33	Recommendation based on review of available studies.	[218]
Sheep	7.36	Depletion/repletion study of BC from corn silage in lambs (*n* = 56); hepatic VA used to indicate VA status.	[123]
Horse	6–10	Recommendation based on review of available studies.	[215]
Horse	>10	Depletion/repletion study with BC supplement in water-soluble beadlet form; serum retinol and relative dose response test used to indicate VA status (*n* = 45). Supplementation with 215,00 *μ*g BC/d did not restore VA status, while 21,500 RE as retinyl palmitate did (though not completely).	[151]

Omnivorous mammals			
Rat	2	Recommendation based on review of available studies.	[156]
	6	Depletion/repletion study using dry gelatin beadlet form of BC as supplement; hepatic VA used to indicate VA status.	[157]
Mongolian gerbil	6–13	Depletion/repletion study using water-soluble beadlet form of BC as supplement (*n* = 80); hepatic and renal VA used to indicate VA status.	[158]
Human	2 in oil 12 in food	Recommendation based on review of available studies.	[166]
Human	2.4	Stable isotope study administering physiological doses of ^13^C-BC in oil to children for ≤10 wk (*n* = 35); concurrent administration of ^13^C-retinyl acetate allowed estimation of BC bioefficacy from serum response.	[75]
Human	9.1	Stable isotope study administering single oral doses of ^2^H-BC to adults (*n* = 22); concurrent administration of ^2^H-retinyl acetate allowed estimation of BC bioefficacy from serum response.	[78]
Human	14.8 in carrots; 20.9 in spinach	Subjects consumed single meal of intrinsically labeled ^2^H-spinach and ^2^H-carrots (*n* = 14); concurrent reference dose of ^13^C-retinyl acetate allowed estimation of BC bioefficacy from serum response.	[77]
Pig	6.7	Recommendation based on review of available studies.	[216]
Pig	13–27	Depletion/repletion study of BC from corn (*n* = 171); hepatic VA used to indicate status.	[124]
Pig	40	Gilts were fed low VA diet for only 4 weeks, and there was no indication that they were truly depleted (*n* = 36); supplemented with 100 mg BC in water-soluble beadlets; hepatic VA used to indicate status.	[169]

Carnivorous mammals			
Ferret	>15	Depletion/repletion study with BC supplement from water-soluble beadlets (*n* = 145); hepatic VA used to indicate status.	[183]

Birds			
Chicken	2	Recommendation based on review of available studies.	[195]
Chicken	6	Depletion/repletion study using dry gelatin beadlet form of BC as supplement; hepatic VA used to indicate VA status.	[157]
Cockatiel	>2	Depletion/repletion study in which chicks born from breeding pairs fed VA-devoid diet consumed VA or BC (synthetic) supplemented diets for 38 d (*n* = 27); hepatic VA used to indicate status.	[198]

**Table 3:** Summary of *β*-carotene metabolism in animal species.

Species	Absorption of intact BC into blood	Accumulation of BC in tissues	Conversion to retinol	References
Herbivorous mammals				
Cows	Yes	Yes, but varies with breed	Yes	[9, 80, 83, 128, 129, 137, 140, 142–146, 223, 224]
Sheep and goats	Yes, but only with high dose	No	Yes	[123, 128, 137, 147, 148]
Other Artiodactyla	No	No (shown in white-tailed deer)	Assumed	[146, 147]
Rabbits	n/a	No	Yes	[130, 147]
Horses	Yes	Yes	Yes	[99, 147, 149–151]

Omnivorous mammals				
Rat	No	No	Yes	[50, 120, 135, 145, 152, 154, 156, 157, 225]
Mongolian gerbil	Yes	Yes	Yes	[134, 158, 159]
Humans	Yes	Yes	Yes	[51, 74, 77–79, 147, 161, 162, 164–166, 168, 226, 227]
Nonhuman primates	Yes	Yes	Yes	[118, 152, 163, 167, 168]
Pigs	Yes, but only with high dose	Yes, but only with high doses	Yes	[124, 125, 147, 155, 169–172]

Carnivorous mammals				
Ferrets	Yes	Yes	Yes (but inefficient)	[82, 92, 118, 178, 182–184]
Canids	Yes, but only with high dose	No	Yes	[147, 173–176, 180]
Felids	Yes, but only with high dose. Wild felids may absorb BC more efficiently than domestic cats	No	Yes (but inefficient)	[18, 27, 47, 130, 177, 185–187]

Birds	No, xanthophylls absorbed preferentially	No	Yes (shown in chickens, cockatiels, canaries, quail, and ducks	[157, 188, 194–200]

Fish	No, xanthophylls absorbed preferentially	No	Yes	[201, 203, 206–209]
